# The Power of a Profound Experience With Nature: Living With Meaning

**DOI:** 10.3389/fpsyg.2022.764224

**Published:** 2022-06-02

**Authors:** Becky Mathers, Eric Brymer

**Affiliations:** ^1^School of Education, Arcadia University, Glenside, PA, United States; ^2^Faculty of Health, Southern Cross University, Lismore, NSW, Australia

**Keywords:** profound nature experiences, significant life experiences, pro-environmental behaviors, wellbeing, meaning

## Abstract

Nature based experiences have been linked to significant positive outcomes for people and the planet. Significant life experience research investigates the associations between formative experiences in nature and resulting environmental concern and action, including both singular events and repeated experiences. However, little is known about the long-term impacts of singular profound experiences with nature. This research sought to better understand how a singular, meaningful experience defines an individual’s self-awareness of his or her relationship with nature, changes social relationships, and directs environmental decisions and behaviors. Twenty-one adults who had a profound experience with nature participated in a semi-structured interview exploring how they make sense of their experience, the meaning they attribute to it, and the role it served in their lives. Three themes resulted from the thematic analysis process: (1) Living in relation with nature, (2) Living authentically, and (3) Living a meaningful life. The findings demonstrate that a single profound experience with nature can have long-lasting and significant effects on an individual. Understanding these long-term influences of a profound experience with nature have implications for intervention designers such as health practitioners and environmental educators, as well as policy-makers.

## Introduction

In recent years there has been an increased interest in understanding motivations and processes that contribute to pro-environmental behavior. Researchers from numerous disciplines have found various factors that influence pro-environmental decisions and behavioral choices such as where to shop, what to buy, how to spend free time, or more significant life decisions related to academic paths, career tracks, or major life shifts. The internal or personal factors include values, beliefs, mindfulness, attitudes, emotions, self-efficacy, moral responsibilities/personal norm, motivation, habits, personality traits and character, worldview, knowledge, demographics (age and gender), childhood experience, sense of control, political views, cognitive biases, place attachment, and chosen activities (Schultz et al., 2005; De Groot and Steg, 2007; Amel et al., 2009). External factors include infrastructure, affordances, social norms, reinforcement contingencies, prompts, feedback and goals, economic constraints, social pressures, comfort and convenience, positive versus negative messaging, time, religion, urban–rural differences, social class, proximity to problematic environmental site, and culture/ethnicity (Clayton and Myers, 2009; Harré, 2011; Quimby and Angelique, 2011).

Insights into how individuals come to value their relationship with the natural world are important as they can help intervention design and policy development. Significant Life Experience (SLE) research, grounded in the field of environmental education, began with Tanner’s (1980) research into the types of formative experiences in nature that led to conservation work as an adult was the catalyst for many future studies. Since Tanner’s (1980) original study, SLE researchers have enlarged and redefined this area of research (Palmer and Suggate, 1996; Chawla, 1998, 1999, 2007; Palmer et al., 1999). This area of research is an important area where scholars investigate how time spent in nature contributes to the preparation of individuals who “take action for the environment” (Chawla and Derr, 2012, p. 527).

The goal of SLE research is to understand the formative nature experiences that are most influential in launching future environmental interests. As the research in this field has developed and expanded over the years, the findings have remained consistent. Participants describe the most influential experiences shaping their environmental attitudes, values, and behaviors, including outdoor experiences during childhood, participating in organized groups, family vacations, trips to environmental education or nature centers, memorable teachers or classes, books, and witnessing the destruction of a beloved natural space. Chawla (1998) summarized the significance of consistent findings when she wrote, “The fact that the same cluster of results emerge under most conditions suggests that, across countries and cultures, people understand the sources of their environmental attitudes and actions in similar ways” (p. 361).

Although scholars have recently begun to investigate a single, memorable experience with nature (Merrick, 2008; Caston, 2014), little is known about the long-term effects of these experiences. This paper, reports on research that seeks to better understand the impact of singular, meaningful experiences. Various terms have been used to describe these profound moments with nature such as peak experience (Maslow, 1964), quantum change (Miller and C'de Baca, 2001), transcendent experience and flow (Csikszentmihalyi, 1997; Williams and Harvey, 2001; Cohen et al., 2010), epiphany (Jauregui, 2007; McDonald, 2008), and environmental epiphany (Merrick, 2008; Vining and Merrick, 2012). In this paper, an experience which shifts an individual’s view of or relationship with the natural world, will be referred to as *a profound experience with nature* or *profound experience*.

Profound experiences with nature encompass both direct and indirect experiences with nature. Direct experiences occur outdoors in a natural setting and indirect experiences include exposure to nature in an abstract way, such as indoors in a classroom, through a film or book, or *via* other forms of media with an environmental theme. While direct experiences with nature are more often reflected in the literature, indirect experiences also instigate changes in environmental perceptions and behaviors (Peters-Grant, 1986, unpublished doctoral dissertation^1^; Gunderson, 1989, unpublished master’s thesis^2^; James, 1993, unpublished doctoral dissertation^3^; Palmer, 1993; Sward, 1996; Chawla, 1999; Merrick, 2008). This paper, part of a larger study on profound experiences with nature, examines the lasting transformational impact of both direct and indirect profound experiences with nature.

## Materials and Methods

### Research Design

Following ethics approval from a US University, participants (*n* = 21) were recruited using purposeful and snowballing recruitment. Participants were recruited through a local environmental commission in central New Jersey, United States and an alumni group. Study participants were asked to share the study with others who might be interested in participating. Participants had to meet the following criteria, 18 years of age or older, self-identify as having had an experience that shifted their view of, or relationship with, the natural world, and be willing to share the effects of their profound experience with nature.

### Participants

All participants but one was college educated and 16 participants had advanced degrees. Ages ranged from 30 to 68, with a mean of 48. Fifteen participants were female, all except one identified as Caucasian, one participant also identified as part of Native American. At the time of the interview, one participant was a student, one was retired, and the remaining 19 participants were working professionals. All participants were living in the United States (Table 1).

**Table 1:** Demographic information (all names are pseudonyms).

Name	Age	Location	Setting
Bridget	57	Cranbury, NJ	Suburban
Tina	52	Cranbury, NJ	Suburban
Joseph	68	Hopewell, NJ	Suburban
Lindsay	32	San Francisco, CA	Urban
Ruth	60	Philadelphia, PA	Urban
Ashley	36	New Hope, PA	Suburban
Tracy	51	Princeton, NJ	Urban
Melanie	51	Hopewell, NJ	Rural
Erin	30	Philadelphia, PA	Urban
Jonathan	56	Palmyra, PA	Rural
Stephanie	44	Roaring Brook Township, PA	Rural
Nicholas	32	Hopewell, NJ	Rural
Michelle	32	Bloomsburg, PA	Suburban
Julie	39	Staten Island, NY	Urban
Susan	64	Hopewell, NJ	Suburban
Kyle	47	Key Largo, FL	Urban/suburban
Hope	48	San Raphel, CA	Suburban
Jeffrey	65	Hatboro, PA	Suburban
Mary	61	Belle Meade, NJ	Rural
Kim	52	Litlitz, PA	Suburban
Chad	40	Dover, NH	Urban

### Data Collection

Twenty-one participants engaged in an individual semi-structured interview consisting of four guiding questions and eight additional questions. The interview protocol asked primarily open-ended questions that “build upon and explore” (Seidman, 2006, p. 15) participants’ responses to encourage their reconstruction of profound experiences with their personal nature stories. The content of the interview questions was meant to elicit both perceived impacts of a profound experience with nature, and was derived from Miller and C’de Baca (2001), Merrick (2008), Caston (2014), and Liddicoat and Krasny (2014).

All interviews were undertaken by the first author. Participants were invited to recount their profound experience with nature with as much detail as possible, describe why they thought this experience was so memorable, reflect on any meaning this experience currently holds and if and how the meaning had changed over time. Participants were also asked if they saw themselves differently than before the experience and to describe the effects of their profound experience with nature. Participants were left to define nature in their own way (see Table 2 for details on the specific nature experiences) and describe the effects of their profound experience with nature. If a participant needed additional prompting, guiding questions were used to solicit more information related to the time of the event, the actual experience itself, or the after effects.

**Table 2:** Overview of experiences with nature participants described as profound.

Bridget	Watching ants collect and carry soil to an anthill
Tina	Reading the book *13 Original Clan Mothers*
Joseph	Seeing baby birds in a nest for the first time
Lindsay	Falling in love with rocks on the side of a volcano
Ruth	Sleeping in a canoe on a lake by herself
Ashley	Driving with her parents and seeing trash on the sides of the highway
Tracy	Encountering a boy who told her she peed in her water
Melanie	Learning to swim at age 49
Erin	Interning on an organic farm
Jonathan	Witnessing the changes in tree species at a scout camp
Stephanie	Playing in abandoned fields near her home
Nicholas	Seeing a Hummingbird in his backyard
Michelle	Working on a wood turtles research project in Nova Scotia
Julie	Camping overnight for the first time
Susan	Seeing an image of nature in a social worker’s office
Kyle	Thru-hiking the Appalachian Trail
Hope	Encountering a giant cuttlefish while scuba diving
Jeffrey	Giving cabbage to three women in a high-rise apartment complex
Mary	Becoming a rock on her grandmother’s farm
Kim	Walking into Mariposa Grove and seeing giant Sequoias for the first time
Chad	Meeting a white-tailed deer while fly-fishing

Each participant was interviewed once. By the end of the interviews, noticeable patterns related to the effects of a profound experience began to emerge. Fusch and Ness (2015) wrote, “Data saturation is reached when there is enough information to replicate the study, when the ability to obtain additional new information has been attained, and when further coding is no longer feasible” (p. 1408). In addition, Holloway et al. (2010) suggest that for data to be saturated, qualitative researchers must allow the data to represent the voices of the participants and not of the researcher. To accomplish this, inductive analysis was used to draw conclusions from participant narratives rather than a predetermined framework, as described in the following section. The themes created weave across all the profound experiences shared during the interviews indicating saturation was reached.

### Data Analysis

To understand the lasting effects of a profound experience with nature Braun and Clarke’s (2006) thematic analysis (TA) was used to identify, analyze, and interpret the data. An inductive approach was used to learn how participants understood the effects of their profound experience with nature rather than assigning themes derived from the literature. To ensure intercoder reliability within the data, two qualitative researchers, both of whom have earned a doctorate and have extensive qualitative research experience, were enlisted as part of the analysis team.

To develop a preliminary interpretation of the data, interesting or meaningful keywords and phrases were identified, and summaries were written to describe each participant’s experience. Each interview was coded entirely before moving onto the next. Previously coded data was continuously reviewed to ensure each new code was unique and no overlap occurred. Any words or phrases that appeared in more than one interview was noted to help with inductive theme development.

After this initial coding, the data was viewed as a whole to begin to establish relationships between codes and combine them into subthemes. For example, while all participants spoke about changes in their relationship with nature, they used different words to describe how it changed, such as connection, interconnection, interconnectedness, one with nature, one with everything around me, a moment of cohesion, or less apart from nature. Rather than having an individual theme for each term, these words were combined under the subtheme of connectedness.

Once initial subthemes were developed, it was necessary to determine if and how they combined naturally into themes in a manner that could describe the effects of the experience. A coding guide was created that included a sampling of inductively created codes and subthemes, and was sent to collaborating coders for review. The coding guide was revised numerous times in consultation with collaborating coders, evolving from a simple interpretation to one of depth, genuinely reflecting the effects described by the participants.

Once it was agreed that the subthemes were distinct, the subthemes were connected into themes that worked across the entire data set. Any overlap between themes was explored, which involved looking at individual themes while also considering how each theme related to the other themes. A composite description was crafted for each theme before assigning it a name, to enable the distinction between various subthemes that comprised each theme. Once the connections between the subthemes were identified, it was easier to name the theme. Throughout this process, the data was revisited to ensure the essence of each effect reported was reflected, using the participants’ own words as much as possible in the composite descriptions. This inductive analysis, conducted collaboratively with three coders, resulted in themes that combine to form a composite story detailing the effects of a profound experience with nature.

## Validity Issues

Credibility was established by soliciting feedback from the participants regarding the interpretation of the effects of their profound experiences with nature. Participants were emailed the final themes and composite descriptions and asked if the themes represent the effects they experienced, and if so, which theme(s) best represented their effects, or if not, how would they describe the influences of the experience. Seventeen of the 21 participants (81%) replied to the request; all of them confirmed that the themes accurately represented the effects of their experience.

Credibility was also established through peer review. Three collaborative coders evaluated the findings. Each had an opportunity to look at pieces of the data and code it individually. Any differences in the interpretations were discussed and negotiated.

To allow for transferability, vivid details of participant’s interviews were provided. Additionally, an open definition was utilized, rather than a more structured one, to allow potential participants to define profound for themselves, thereby opening the study to more variety among experiences. To address dependability, an extensive description of the methods and research context are included. To achieve confirmability, the data was repeatedly revisited, thereby allowing the themes to emerge naturally rather than making the data fit into pre-existing themes.

## Results

This study set out to investigate how profound experiences with nature facilitate pro-environmental behavior. The experiences shared by the participants illustrated the diversity of experiences considered profound (Table 2). A pseudonym is used for each participant throughout this paper.

A profound experience with nature was the impetus for change for every participant in this study. Participants often consider the consequences of their choices and contemplate how their decisions impacted themselves, others, and the planet. Three themes *resulted from the analysis process* (1) Living in relation with nature, (2) Living authentically, and (3) *Living a meaningful life.*

### Living in Relation With Nature

Participants described how their profound experiences influenced their shopping habits, resource use, and free time. Participants described being more mindful about where their food comes from, buying locally where possible, reducing food waste, and building a relationship with food producers. For example, Erin reflected that her profound experience from her time on an organic farm meant that she was now “obsessed with buying local food, and I love going to the farmers’ markets and talking with the farmers.”

Participants described altering resource use after a profound experience with nature, most often this included recycling, composting, reducing convenience behaviors and actively attempting to care for animals and the planet. For example, Julie spoke of a camping trip that resulted in her making a shift from convenience behaviors (using a plastic water bottle and throwaway coffee cups) to more mindful behaviors such as composting. Likewise, Stephanie became passionate about changing her lifestyle and encouraging others to support the sustainability of the planet. Susan reported actively looking for opportunities to be pro-environment, while also recognizing the need to do more. She stated,

“I feel reverent about the environment. When I’m on my walks, if I see trash or garbage, I’ll pick it up and I’ll save it until I get out and have a place to recycle it, and I recycle here at home. I try to conserve energy.”

Participants also reflected on how their experience altered their choice of free time activities. These changes included engaging in more outdoor activities, reading about environmental topics, volunteering for environmental organizations, and becoming more politically active.

Participants reported that their profound experience initiated an active search for opportunities to spend more time in nature. Susan described seeking refuge in nature, which began with that first image of a calming nature scene. When dealing with issues related to her son she said, “My means of coping with his very abnormal development was to walk and walk and walk and walk and walk, miles and miles a day and just soothe myself with nature.” Melanie craves more time in aquatic environments after losing her fear of water and learning to swim. She said, “It’s like I’ve got to have water. I did not think of it as something I needed and so now whenever we make plans or have a special treat it usually involves water.” Ruth sees her experience of sleeping on the canoe as a “catalyst for other moments” and outdoor adventures she pursued in her life. She described participating in an Outward Bound program, becoming a canoe instructor, studying with a boat builder and building her own canoe, and venturing to a remote part of India to learn about the relationship between spirituality and the forest.

Other participants have engaged in activities such as political involvement or learning more about the environment. For example, after reading *13 Original Clan Mothers*, Tina reads more about the natural world and volunteers for her town’s environmental commission. Kim has taken a heighten interested in politics as demonstrated by her comment, “I think that I’m more aware and pay more attention politically to environmental aspects of decision-making—in the community and also further around across the United States.”

### Living Authentically

Individuals recounted how their profound experience helped them gain a better understanding of who they are, and become more aligned with their true nature, core values, and beliefs. Authenticity manifested in three ways: developing a sense of empowerment, reprioritizing values, and expanding spiritual beliefs.

#### Sense of Empowerment

Participants recounted feeling a new sense of self-agency after having a profound experience with nature. Participants used words such as “proud,” and “validation,” or “turning point,” and “you can do anything,” or “you can learn anything,” to describe how their profound experience instilled in them the confidence and courage they were previously missing. For example, Erin felt proud for deciding to intern at an organic farm after completing her undergraduate studies despite pressure to find a high-income job.

Similarly, Jonathan’s confidence in his ability to apply what he was learning in his undergraduate education was solidified after seeing the transition of tree species in the forest. He stated that his experience “was just very transformative for me because it was a validation of my education, which was exciting… suddenly gaining the confidence in my own skills.”

One participant discovered the ability to be comfortable on her own in the natural world. Michelle had never liked to be alone, but while participating in the turtle research project, Michelle would spend 10–14 h a day outside by herself. She reflected on how it affected her, “I had to learn to be alone… I feel like that was a big turning point, learning that I could be by myself and be okay. Be alone with my thoughts.”

For some participants, a profound experience with nature facilitated self-assurance, and confidence in their strengths and their capacity to succeed. For example, Ruth gained self-confidence after sleeping alone in the canoe. She remarked,

“I think there’s certain things that help you to feel like you can do anything that you want to do and that there are no obstacles… to know the importance of being alone in nature and knowing that I could be alone in nature would be okay.”

Julie’s first overnight camping trip is the basis for her sense of self. She stated, “I just kind of feel like it somewhat defines me as a person… It’s like, I had this experience. I’m richer for having that. This is part of me now.”

Learning to swim at the age of 49 gave Melanie the confidence to try other things such as playing the piano. “It literally showed me that you can learn anything or change something that you think is pretty deeply entrenched at any time and that is a really good thing to know.”

Participants described how their profound experiences triggered comprehensive life changes. For example, Susan attributed her ability to escape emotional abuse at home to her profound nature experience. Nicholas’s profound experience watching a hummingbird hovering, triggered his decision to “work on” his alcohol consumption.

#### Reprioritizing Values

Two experiences described in this study also prompted individuals to consider and reprioritize their values or guiding principles, placing more priority on nature and less on themselves. For example, Jeffrey’s experience changed his priorities.

“This is gonna start with what I think of as ego. For me, my sense before this [experience], my sense of value, my sense of worth came from what I accomplished, what my legacy was. What came out of this was that was completely wrong. My sense of value, your sense of value, every human being’s sense of value, the mere fact that they were born, they have intrinsic value.”

#### Expanding Spiritual Beliefs

For some participants’ a profound experience shifted their sense of truth and certainty about the possibility of some higher existence or meaning in life. Participants who spoke of this expressed how before the experience, they considered themselves not very religious or spiritual or for some, even agnostic. After a profound experience with nature, these participants felt open to a belief in something more.

When Hope made eye contact with a giant cuttlefish, it challenged her disbelief in a higher power. She reflected, “But it was this experience of …I felt like I was staring into the face of God.” She reiterated how the experience reinforced an underlying belief she had. She continued,

“But this cuttlefish was just so remarkable. And it created just a sort of a belief in some … Not in any kind of Jewish or Christian or Muslim God, or any other God. It was nothing like that. It was really just this sort of reminding me of my deep belief in just the healing nature of the earth.”

As Kim walked among the giant sequoias, she acknowledged the possibility of a greater power. She recognized her place in the world and the significance of all life:

“I was never really religious before and I think that when you see something like that it makes you realize that there is the possibility of something else out there that is controlling things… and whatever you call your God or whatever, Mother Nature… It was just absolutely amazing and spiritual. It made me realize how insignificant humans are and that nature keeps on going whether we are here or not.”

Likewise, Mary’s experience on the rock helped her understand spirituality. She reflected; “the Rock taught me so much. It taught me what God meant… spiritually you felt supported and safe and loved.”

Jeffrey’s spiritual beliefs increased after giving cabbage to the Korean women. He acknowledges that the experience saved him emotionally and spiritually. “It’s a spiritual thing. It’s healing. It’s healing for me personally.”

### Living a Meaningful Life

Participants also noted that a profound experience with nature triggered meaningful life changes, whether related to an academic path, a career track, or a more significant life transformation. This directional life change enabled the participants to live a life that felt truer and more authentic. For example, Bridget attributed her experience with the ants to the academic path she chose more than a decade later. Standing in her backyard, Bridget became absorbed by the sight of ants transporting individual particles of soil in their mouths as they built anthills. Fascinated by the work ethic of the ants and their connection to the soil, Bridget felt awe and in that moment recognized the interconnectedness of all living creatures. She reflected, “It’s kind of made a trajectory of me and nature; And I studied it, I had my PhD in it and … it kinda has set me into like this particular direction.”

Tracey attributed her return to law at school at 49 to her profound experience with nature. She stated, “It was after this boy had said this [‘Are you American?… Oh. You’re rich. You pee in your water.’], several years after, that all of a sudden it kind of came back to me. And I was like, Hm, this is why I need to make a change.”

Hope reflected that her experience with a giant cuttlefish, was behind her decision to study for a doctoral degree in Jungian Psychology. After her experience of feeling like she was staring into the face of God, she stated, “I think that because of this sort of experience of God, so to speak, I was more open to go into a school that was Jungian focused.” Chad believed his decision to attend a particular graduate program stemmed from his encounter with a deer. On a fly-fishing trip, Chad saw a small buck near the water. He wanted to capture the moment with his camera and slowly moved closer to the deer. When Chad was within a few feet of the deer, he and the deer made eye contact and stared at each other for a long time. Something inside Chad urged him to get even closer. The buck, instead of running away as Chad expected, stepped closer to Chad. The two were now arms-length apart and Chad instinctively reached out his hand and touched the deer’s snout. Chad felt connected to the deer as they stared deeply into each other’s eyes. The young buck leaned forward and licked Chad’s hand with his rough tongue. He stated,

“The time that I read that [program description] was after that deer experience. I think I gathered from the description of ETL [Ecological Teaching & Learning] that that was the magic that I had experienced with the deer experience and I wanted that. So yeah, I think it has somewhat influenced my path in life.”

A profound experience with nature also affected career choices. For some the experience initiated a nature specific career choice such as working in environmental fields or as a Park Ranger. For others the choice was less specific but involved the desire to “make a difference” (Michelle). For example, seeing trash along the highway was the impetus for Ashley’s future career in land stewardship. She reflected,

“Now it’s starting to come to full fruition if you think about it. Now I’m stewarding the environment trying to remove the trash and the unwanted items, or making it more ecologically sound for the good of all of the creatures.”

While Mary was reflecting on her profound experience with the rock, she made a connection between the event and her future career as a physical therapist. She remarked,

“I’m also a musician, but when I made the decision to become a physical therapist, I needed something that I could support myself with and I said, “People may love the flute, but they are not always going to pay for it, you cannot make a living on it.” So, I said I needed a rock-solid career, so I can use in my language. To be a physical therapist was like a rock because people are always going to get sick and I would always have business.”

## Other Findings

### Age at Time of a Profound Experience With Nature

The age at which participants reported having a profound experience with nature varied from early childhood through late adulthood (Table 3). The mean age is 25, with a range of 6–63. The age range during which the greatest number of participant’s report having had a profound experience with nature is childhood (ages 6–10). The five participants whose profound experience with nature occurred during this age group were engaging in “direct, informal, and unstructured” exploration in natural spaces (James et al., 2010). Bridget was in her backyard, watching ants collect tiny pieces of soil. Joseph was playing hide-and-seek outdoors when he discovered the bird’s nest. Stephanie was exploring abandoned lots and engaging in fantasy play during her profound experience. Mary became the rock when alone in the farm field near her grandparent’s house. Ashley witnessed the harmful behaviors of humans toward nature through a car window while traveling along a busy highway.

**Table 3:** Age range at time of profound experience with nature occurrence.

Age at time of experience	Number of participants	Percent of total
Birth–5	0	0
6–10	5	23.8
11–15	1	4.8
16–20	4	19
21–25	2	9.5
26–30	3	14.3
31–35	0	0
36–40	2	9.5
41–45	2	9.5
46–50	1	4.8
51–55	0	0
56–60	0	0
61–65	1	4.8
66–70	0	0
**TOTAL**	**21**	**100**

The age group with the next highest number of profound experiences with nature occurrences was 16- to 20-year-olds. Four participants recounted experiences occurring during this time. Ruth paddled a canoe into the middle of the lake and slept under the stars. Jonathan recognized changing tree species while at scout camp. Michelle spent up to 12 h a day on her own on a beach in Nova Scotia monitoring wood turtle nests and Julie experienced her first overnight camp while in a teenage ecology workshop. These experiences fall under stage three or four of James et al.’s (2010) development model for becoming an environmentally involved individual. These participants were engaged in outdoor activities with individuals outside of their immediate family and embraced their identities as outdoor enthusiasts or future professionals.

Five of the participants recounted having a profound experience in their 20s, again consistent with James et al.’s (2010) fourth stage of the developmental model. Not one profound experience occurred during participants’ early 30s, yet two experiences happened in the late 30s, and another two occurred in the early 40s. One participant recalled having a profound experience in her late 40s, and while no profound experiences with nature were reported in the decade of the 50s, one participant reported a profound experience with nature after the age of 60.

### Number of Years Since a Profound Experience With Nature

The number of years since having a profound experience varied greatly among participants (Table 4). Only about a quarter of the experiences shared in this study happened fewer than 10 years ago (23.8%). The majority of the experiences happened more than a decade ago (76.2%).

**Table 4:** Years passed since profound experience with nature occurrence.

Number of Years	Number of participants	Percent of total
0–10	5	23.8
11–20	6	28.5
21–30	3	14.3
31–40	2	9.5
41–50	3	14.3
51–60	1	4.8
61–70	1	4.8
**TOTAL**	**21**	**100**

### Location of a Profound Experience With Nature

Study participants reported having a profound experience with nature in a variety of locations. Six participants (28.6%) reported having their profound experience with nature in an indoor location, while 15 participants (71.4%) recounted having their experience in an outdoor location. The variety of locations indicates that these experiences can occur both indoors and outdoors, supporting my decision to include both direct and indirect experiences as demonstrated in Table 5.

**Table 5:** Reported locations of participants’ profound experiences with nature.

Bridget	In the backyard of her childhood home
Tina	Inside her home
Joseph	In a field beside his childhood home
Lindsay	On a small hill near the visitor’s center on Mauna Kea
Ruth	In a canoe on a lake at Camp Louise in northeastern PA
Ashley	In the backseat of her parent’s car driving between NJ and PA
Tracy	Outside in a small village in Africa
Melanie	In a swimming pool
Erin	On an organic farm in Bucks County, PA
Jonathan	In a Boy Scout camp in MD
Stephanie	In the woods near her childhood home
Nicholas	In his dining room looking out into the backyard of his home
Michelle	On a beach in Nova Scotia
Julie	In the Adirondacks
Susan	In a social worker’s office
Kyle	On the Appalachian Trail
Hope	In the water off of Borneo
Jeffrey	In a high-rise apartment building in Trenton, NJ
Mary	On a boulder in a field in VT
Kim	In Mariposa Grove
Chad	Next to the Colorado River in NH

## Discussion

Profound experience with nature were investigated for this study. This research demonstrates that a profound experience with nature can influence an individual’s understanding of self, social relationships, and subsequent decisions and behaviors.

Below, we situate the findings with respect to the literature, theory, and research most relevant to my study. We begin by discussing the choice of a new term, “a profound experience with nature.” Next, we organize the discussion in terms of the three identified themes. Then, we address the implications of this study, as well as the limitations and biases encountered during the course of the research. Finally, we offer suggestions for future research in hopes of acquiring greater insight into the lasting effects of a profound experience with nature.

### Choice of the Use of an Umbrella Term

This research focused on singular profound experiences with nature, which is different from other SLE research. While SLE research does include these single experiences, it also includes repetitive or extended experiences. This study is therefore unique in its the focus on singular profound moments. The term used to describe these singular moments, which shift an individual’s view of or relationship with the natural world, has precision to it in that it focuses on just this one type of SLE. In this research, we discovered there can be great variety among the experiences that participants describe as profound. This diversity in profound experiences demonstrates the need for a term that focused on singular profound moments as a subset of SLE research.

The term “a profound experience with nature” was decided upon because it was inclusive of similar phenomena studied in the past while remaining open to the possibility of yet-unexplored experiences. We recognized and appreciate that profound can mean something different to each one of us. What is most important is that the study participants reported that the experiences they shared were profound.

Participants interpreted the term “profound” differently, yet it became evident during data analysis that similarities existed across the experiences. Participants described the experiences as: personally transformative, illuminating, meaningful, emotional, memorable, aesthetic, unexpected, fleeting, enlightening, enduring (in results), ineffable, leading to the realization of one’s true identity, providing a sense of transcendence, and passive. Similarly, emotions related to a profound experience described by participants included: happiness, awe, ego-transcending, connection, and loss of fear and anxiety. While not every profound experience described in this study encapsulated each of these characteristics or emotions, these were all mentioned by one or more participants.

## Discussion of Major Findings: Three Emergent Themes

### Living in Relation With Nature

For all 21 study participants’ profound experiences with nature inspired decisions and changes in behavior. Participants described using the experience to guide everyday decisions such as where to shop, what to buy, or how to spend free time, or as inspiration for more significant life decisions related to academic paths, career tracks, or major life shifts. The SLE literature abounds with studies indicating a connection between formative experiences in nature and resulting care, concern, and action toward the environment [Tanner, 1980; Peterson, 1982, unpublished master’s thesis^4^; Peters-Grant, 1986, unpublished doctoral dissertation (see footnote 1); Gunderson, 1989, unpublished master’s thesis (see footnote 2); James, 1993, unpublished doctoral dissertation (see footnote 3); Palmer, 1993; Sward, 1996; Chawla and Derr, 2012; Liddicoat and Krasny, 2013]. Likewise, previous studies of related experiences document a transition to more environmentally beneficial behaviors and activities (Merrick, 2008; Brymer et al., 2009; Caston, 2014; Davis, 2016). Findings in this study are consistent with the results described above, as evidenced by participants recounting changes in behaviors considered beneficial to the environment including in their daily mundane decisions, time spent outdoors, academic pursuits, and career paths.

Positive behavior changes such as buying local products, purchasing organic or GMO-free food, reducing food waste, composting, driving an electric vehicle, using a reusable water bottle, recycling, picking up trash, and caring for plants and wildlife were described by eight participants. Likewise, nine participants spoke about engaging in more nature-based activities such as walking outdoors, canoeing, backpacking, reading, and volunteering after their profound experience with nature. This finding demonstrates a connection between single profound experiences and behavior change, signaling that fostering similar experiences may help encourage better environmental behaviors in others.

### Living Authentically

The vast majority of participants felt more authentic after their profound experience suggesting that these types of experience have the potential to redefine an individual’s understanding of self. Participants gained self-confidence or courage following their profound experience, empowering them to define their own sense of identity. A similar effect has been documented in previous research that describes individuals who feel more in tune with themselves during a moment of profundity such as a peak experience (Maslow, 1964) or spiritual transformation (Cohen et al., 2010). Many commonalities exist between these experiences, including the characteristics of being memorable, illuminating, involving profound beauty, and experiencing a sense of transcendence leading to personal transformation and a realization of one’s true potential. People who undergo these experiences express feelings of happiness, wonder, peace, and a connection to something greater than self. Overall, these experiences are positive and allow individuals to feel capable, confident, and empowered to be a better version of themselves.

Becoming authentic also manifested as a shift in personal values from a focus on personal success to place more emphasis on the natural world. From an environmental psychology perspective, this reflects a transition from *egoistic values*, associated with individual concerns or outcomes, to *biospheric values*, which are values more aligned with nature (Stern et al., 1993; Steg et al., 2005; De Groot and Steg, 2007, 2008).

Previous studies of related phenomena also describe similar findings. While Merrick (2008) did not specifically isolate value changes after an environmental epiphany, she reported that 75% of her study participants described changes in environmental values, attitudes, and behaviors. Similarly, Miller and C'de Baca (2001) reported lasting changes in attitudes, beliefs, emotions, and values resulting from quantum change. Maslow (1964) also documented cases of individuals becoming less selfish and transcending the ego while in a peak experience. While findings from this study confirmed the potential of a value shift after a single profound experience, only two of the 21 participants reported this effect, which is significantly less than that reported by Merrick (2008). However, participants were not explicitly asked if their values changed because of their profound experience. Hence, this finding warrants a more in-depth exploration between the potential connection of profound experiences with nature and subsequent value changes.

Becoming authentic was also described as an expansion of spiritual beliefs or an acceptance of a universal truth. Profound experiences triggered an openness to a belief in something “more” such as the possibility of a higher power like a God, Gaia, or the healing power of nature. These characteristics are discussed extensively in terms of conversion and mystical experiences (James, 1929), peak experiences (Maslow, 1964), quantum change (Miller and C'de Baca, 2001), transcendent experience (Roy, 2001; Williams and Harvey, 2001; Davis, 2016), awe (Maslow, 1964; Keltner and Haidt, 2003), epiphany (Jauregui, 2007; McDonald, 2008), and spiritual experiences (Cohen et al., 2010). Each of these experiences offers insight into new truths or meanings, leading to personal transformation. This expansion of spiritual beliefs and opening to the possibility of universal truth are essential to feeling connected to something greater than self.

Five participants discussed how they felt a deeper connection to everything else in the universe resulting from their profound experience with nature. They described it as an interconnectedness between all living beings and the nonliving world, which participants viewed as no longer feeling apart from nature but instead a part of nature. Participants used this intimate connection to understand their place in the universe, resulting in a new sense of self, as part of nature. These findings support the results of similar studies. Williams and Harvey’s (2001) study of transcendent experiences found that participants expressed harmony with the greater world, and outdoor recreationists in Brymer et al.’s (2009) study also reported feeling connected to the natural world in which they practiced their extreme sports. Similarly, Perrin et al. (2018) reported that participants felt a stronger connection to and more profound respect for the natural world after an encounter with a wild animal. These findings suggest that profound experiences have the potential to expand an individual’s conception of self beyond the physical limitations of the human body into the greater universe.

### Living a Meaningful Life

The influence of profound experiences with nature on academic choices most often ignited or strengthened a passion for the environment and studies on the human dimensions of natural resources, soil science, environmental law, and ecological teaching and learning. Likewise, the profound experience with nature either strengthened an existing career path or was the inspiration for a career change.

Interestingly, a profound experience with nature did not necessarily lead to a career dedicated to environmental care or protection, as demonstrated by two participants who followed another path (clinical psychology and physical therapy). During the interviews, both participants acknowledged a previously unrealized connection between their profound experience and their career. Hope’s experience with the giant cuttlefish triggered within her a sense of wholeness and connection with the world. It prompted her to consider her relationships more closely, consider her existence, and examine the decisions she made. Years later, she finds herself studying Jungian Psychology and recognizes the connection between her profound experience and her career choice. She stated, “I cannot help but think that there’s something about this experience that made me choose the PhD program I went to… And I think that because of this sort of experience of God [when seeing the cuttlefish]…I was more open to go into a school that was Jungian focused.” Similarly, as Mary acknowledged that her decision to become a physical therapist was because her time with the rock led to her pursue a “rock-solid” career. She reflected, “To be a physical therapist was like a rock because people are always going to get sick and I would always have business.” These findings challenge scholars to think more deeply about the underlying connections between profound experiences and resulting academic and career decisions. While it makes sense on the surface that an experience that shifts an individual’s view of or relationship with the natural world has the potential to lead to an environmentally-focused career, what may have occurred is a shift in one’s values or priorities, ultimately leading to a career more-aligned with these new personal principles.

For two participants in the present study, their profound experience with nature was life changing. The moment signified an escape from a negative environment or destructive behaviors and the opportunity to begin anew. For both, they recognized the significance of the moment and how their lives would never be the same. Their profound experience with nature significantly changed their behaviors, their entire life course, and their understanding of who they are and could become. It should be noted that these life-altering affects are not exclusive to profound experiences with nature. Previous findings (James, 1929; Miller and C'de Baca, 2001; McDonald, 2008) affirm the potentially life-changing effects of profound-like experiences including a profound experience with nature.

## Limitations of the Study

Research in the field of SLE such as this study have numerous strengths as outlined by Chawla (1998). One strength is the qualitative nature of the research allowing scholars to explore “the emotional and interpretive side of environmental experience” to fully grasp the entirety of it (p. 361). This qualitative work includes the use of open-ended methodologies, which allow respondents the time to consider their responses. It often employs a lifespan perspective as participants are asked to recall formative experiences in nature that may have occurred decades ago. With almost 40 years of research, the SLE field has grown and expanded from Tanner’s (1980) pioneering research. Studies are becoming more diverse and focusing on women, as well as people from different countries, cultures, and ethnic backgrounds (Chawla, 1998). There is a new interest in understanding how individuals of varying age groups interpret their formative experiences in nature, and efforts have been made to design longitudinal studies.

While the strengths of SLE research are numerous, the field also has areas in which it can improve. By using a purposeful selection process, it was more likely the participants would be inclined toward nature, increasing the potential for them to have a profound experience with nature. The research design and questions were constructed to investigate only those singular and memorable experiences with nature that shifted an individual’s view of or relationship with the natural world and did not seek out participants who spent time in nature but did not have a profound experience.

Another limitation of the study is lack of diversity. While the study included diversity in professional careers, it lacked diversity in other ways. All the participants identified as Caucasian, except for one who described herself as part Native American. The lack of gender, racial, and ethnic diversity limits the ability to learn about the effects of a profound experience on individuals of diverse demographic backgrounds. Recruiting from the general public or targeting specific demographic groups would have increased the potential for greater diversity in the study sample.

While in-depth interviews allow for delving deeper into responses and asking follow-up questions, interviews are laborious and as a result, some potential participants may have chosen not to participate in the study. Utilizing a questionnaire to collect data on profound experiences and the subsequent effects is one way to accommodate potential participants who may shy away from in-person interviews or have time constraints and are unable to commit to a sit-down interview.

All 21 participants recalled a past profound experience with nature and shared the memory and subsequent effects. Using personal memories is a limitation of this study as concerns over the validity of memory to recall past events exist (Ross, 1997). Memory can be flawed, and individuals can remember memories differently at various points throughout their lives. Exploring the effects of a profound experience through an interview may have affected what the participants shared. Participants may have felt the need to answer questions quickly, rather than taking time to reflect on how to answer deeply. One way to account for this limitation is to provide the interview questions in advance, giving the participants time to reflect upon their answers before the interview.

## Further Research Directions

This study provided initial insight into the lasting effects and memory functions of a profound experience with nature. With this new knowledge, new questions also arise. More research is needed to understand the potential benefits of this phenomenon.

Individuals from diverse demographic backgrounds are underrepresented in studies of profound experiences, an area of research that needs exploration to learn how individuals from these unique backgrounds understand the effects of such experiences. Future studies would benefit from incorporating the perspectives of underrepresented populations such as Hispanic Americans, African Americans, Native Americans, Indian Americans, as well as international participants. Equally important to explore is how class, gender, and rural versus urban upbringing affect how participants come to understand the meaning and effects of their profound experiences.

Similarly, the role of culture should be investigated because what self means and the boundaries between self and the natural world can vary greatly from culture to culture. Thus, understanding the cultural background of a participant can elucidate what an individual considers profound, as well as how that an individual understands and describes the effects of a profound experience.

Future studies could also investigate the conditions associated with having a profound experience with nature, such as the role of place, age, or solo versus group activities to understand what makes it the right “time” for the occurrence of a profound experience with nature. Upon reviewing my data, no specific condition “jumped out” as significantly determining the likelihood of having a profound experience. Ten of the 21 experiences occurred with the participant was engaged in a solo activity, while 11/21 occurred during a group event. Similarly, ten of the individuals were under the age of 20 at the time of their profound experience, and 11 were at least 21 years of age. The majority of the profound experiences (61.9%) happened in a place or during an activity familiar to the participant, indicating that novelty may not necessarily be an essential contributor to a profound experience. These findings suggest the need for more extensive studies to investigate what, if any, factors affect the likeliness of the occurrence of a profound experience with nature.

Similarly, future research can explore the potential of having a profound experience after participating in structured outdoor nature experiences provided by organizations such as Outward Bound or Natural Habitat Adventures. A key component of these experiential programs is an emphasis on slowing down, being present in the moment, and taking time for deep reflection. Future researchers can investigate the role of purposeful reflective time in an individual’s understanding of the effects of a profound experience, as well as how these types of structured nature experiences affect the likeliness of the occurrence of a profound experience with nature.

Another interesting direction for future scholars is to investigate the role of psychedelic drugs such as psilocybin (magic mushrooms or magic truffles), lysergic acid diethylamide (LSD), or N,N-Dimethyltryptamine (DMT) on the occurrences or effects of a profound experience with nature. Researchers can investigate the potential enhancing effects of micro-doses of these drugs (dosages around one tenth of the recreational or hallucinogenic dose) on a profound experience (Prochazkova et al., 2018).

Of utmost importance for future research is to further investigate the conditions under which a profound experience with nature is likely to occur. Caston’s (2014) phenomenological study of the dynamics of transformative experiences with nature explored what occurs “within the mind and heart of a person as the experience unfolds” (p. 127). Caston (2014) identified the essential qualities of transformative experiences and the antecedents that influence how an individual perceives the experience. Research is needed to understand the conditions of the experience itself that may affect the likeliness of the occurrence of a profound experience. This information could be used to inform environmental and outdoor educators who strive to craft programming that promotes positive environmental awareness and behaviors, similar to those experienced by individuals who had a profound experience with nature.

## Conclusion

This study provides preliminary insight into the lasting effects of a single profound experience with nature. The findings illustrate that a single profound experience with nature can have long-lasting and significant effects on an individual. Specifically, a profound experience has the potential to encourage an individual’s sense of self, define an individual’s self-awareness about his or her relationship with nature and others, and promote environmental decisions and behaviors. The findings make a valuable contribution to this area of research by demonstrating that SLEs do not require an extended period of time engaged with nature, but that a singular, memorable experience with nature *also* has the potential to shape an individual’s relationship and behaviors toward the environment. Similarly, the findings illustrate that indirect as well as direct experiences with nature can be profound.

This research demonstrates the power of a profound experience with nature. The 21 participants expressed the importance of their profound experience with nature in their life and acknowledged its presence and long-term effects. This research offers new insight into how a profound experience with nature can encourage a new understanding of self, redefine relationships with others and nature as a whole, and promote meaningful and intentional decisions and behaviors.

## Data Availability Statement

The datasets presented in this article are not readily available but potentially identifiable. Requests to access the datasets should be directed to BM, bmathers@antioch.edu.

## Ethics Statement

The studies involving human participants were reviewed and approved by the Antioch University Ethics. Participants provided their written informed consent to participate in this study. Written informed consent was obtained from the individual(s) for the publication of any potentially identifiable images or data included in this article.

## Author Contributions

BM lead the research. All authors contributed to the article and approved the submitted version.

## Conflict of Interest

The authors declare that the research was conducted in the absence of any commercial or financial relationships that could be construed as a potential conflict of interest.

## Publisher’s Note

All claims expressed in this article are solely those of the authors and do not necessarily represent those of their affiliated organizations, or those of the publisher, the editors and the reviewers. Any product that may be evaluated in this article, or claim that may be made by its manufacturer, is not guaranteed or endorsed by the publisher.

## Footnotes

^1^Peters-Grant, V. M. (1986). *The influences of life experiences on the vocational interests of volunteer environmental workers* (Unpublished doctoral dissertation). University of Maine, Orono.

^2^Gunderson, K. I. (1989). *The state of environmental education in Montana public schools*, K–6. (Unpublished master’s thesis). University of Montana, Missoula, MT.

^3^James, K. (1993). *A qualitative study of factors influencing racial diversity in environmental education* (Unpublished doctoral dissertation). University of Minnesota, Minneapolis, MN.

^4^Peterson, N. (1982). *Developmental variables affecting environmental sensitivity in professional environmental educators* (Unpublished master’s thesis). Southern Illinois University at Carbondale.
